# Announcing the 2019 *Medicines* Travel Award for PostDocs

**DOI:** 10.3390/medicines6010030

**Published:** 2019-02-21

**Authors:** Gerhard Litscher

**Affiliations:** Editor-in-Chief of *Medicines*, Head of the Research Unit for Complementary and Integrative Laser Medicine, of the Research Unit of Biomedical Engineering in Anesthesia and Intensive Care Medicine, and of the TCM Research Center Graz, Medical University of Graz, 8036 Graz, Austria; gerhard.litscher@medunigraz.at; Tel.: +43-316-385-83907

For the *Medicines* Travel Award 2019, we received a total of 38 applications from all over the world. The quality of the applications was again very high. After preselection from the managing editorial staff of *Medicines*, a scientific decision was reached by four experts from America (two), Asia and Australia and by the editor-in-chief of *Medicines* from Europe. Therefore, the jury included experts from four different continents again.

As Editor-in-Chief of *Medicines*, I am very pleased to announce the winner of the *Medicines* Travel Award 2019. The travel award was granted to Dr. François Chassagne, PharmD, a postdoctoral research fellow at the Center for the Study of Human Health, Emory University, Atlanta, GA, USA. The award consists of 800 Swiss Francs to attend any academic conference during 2019.


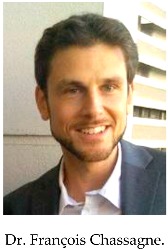


Dr. Chassagne performs laboratory work on medicinal plants, especially cytotoxic, antibacterial and quorum quenching assays against ESKAPE pathogens (an acronym encompassing the names of six bacterial pathogens commonly associated with antimicrobial resistance) under the direction of Prof. Dr. Cassandra L. Quave at Emory University. He also worked as a postdoctoral fellow at IRIT (Informatics Research Institute of Toulouse) in France. Dr. Chassagne published a total of 8 articles in peer-reviewed international journals (including 6 as first author) with over 25 co-authors. Thirty-five citations are reported since 2011. He also serves as a reviewer in renowned journals.

His research objective is to solve health problems by looking for new therapeutic solutions from traditional remedies, especially medicinal plants. He has performed ethno-medicinal studies in Southeast Asia (Cambodia, Lao, Vietnam) and learned how to collect data from traditional indigenous medical systems. This expertise is of utmost importance in the identification of medicinal species with great potential for drug discovery. In addition, he has performed an analysis of chemical compounds from medicinal plants by using traditional and metabolomic approaches and learned how to deal with the high number and variety of compounds found in natural products. Furthermore, he has performed in vitro antimicrobial and anticancer assays of medicinal plants and learned how to validate the traditional use of these natural remedies. He also learned how to use informatics tools in the field of natural product-based drug discovery. The skills were developed during his PhD in France.

Congratulation to Dr. François Chassagne on behalf of the intercontinental jury and the *Medicines* editorial and publishing teams! 

